# Assessment of Menstrual Hygiene Practices and Their Determinants Among Adolescent Girls in Urban Slums of India: A Community-Based Cross-Sectional Study

**DOI:** 10.7759/cureus.90505

**Published:** 2025-08-19

**Authors:** Shubhanshu Gupta, Anshika Agarwal, Amita Sharma, Puneet Agrawal

**Affiliations:** 1 Department of Community Medicine, Government Medical College, Datia, IND; 2 Department of Obstetrics and Gynecology, Government Medical College, Datia, IND; 3 Department of Pediatrics, Government Medical College, Datia, IND

**Keywords:** adolescent girls, maternal education, menstrual hygiene, sanitation, urban slums

## Abstract

Objectives

This study aimed to evaluate the menstrual hygiene practices of adolescent girls in urban slums and examine the socio-demographic and knowledge-related factors associated with good hygiene behavior.

Methods

A community-based cross-sectional study was conducted among 400 adolescent girls aged 10-19 years in selected urban slums of Datia, Madhya Pradesh, India, using multistage random sampling. Data were collected using a pre-tested, interviewer-administered questionnaire. Descriptive statistics, Chi-squared tests, and multivariate logistic regression were performed for data analysis. A p-value of <0.05 was considered statistically significant.

Results

Sanitary pads were the preferred absorbent for 328 (82.0%) participants, with 246 (61.5%) changing them three or more times daily. Soap was used for cleaning by 188 (47.0%), and 224 (56.0%) followed safe disposal practices. School absenteeism during menstruation was reported by 150 (37.5%). Awareness before menarche was present in 230 (57.5%), and 172 (43.0%) cited their mothers as the primary source of information. Good hygiene practices were significantly associated with maternal education (χ²=22.18, p<0.001), private toilet access (χ²=14.27, p<0.001), pre-menarcheal awareness (χ²=10.14, p=0.001), and school attendance during menstruation (χ²=4.21, p=0.040). In multivariate analysis, maternal education (aOR=2.63; 95% CI: 1.59-4.34; p<0.001), pre-menarcheal awareness (aOR=1.91; 95% CI: 1.23-2.98; p=0.004), and private toilet access (aOR=2.08; 95% CI: 1.31-3.30; p=0.002) remained significant independent predictors. School-going status (aOR=1.48; 95% CI: 0.96-2.29; p=0.072) was positively associated but did not reach statistical significance.

Conclusion

Menstrual hygiene practices among adolescent girls in urban slums remain suboptimal and are influenced by socio-educational and infrastructural factors. Promoting maternal and adolescent education, increasing awareness before menarche, and improving access to private sanitation are key to enhancing menstrual health. Targeted interventions addressing these determinants are crucial to ensuring safe, dignified menstruation and advancing adolescent health equity in urban slum settings.

## Introduction

Menstruation is a natural physiological process that marks a girl's transition into womanhood and is a vital component of reproductive health and overall well-being. Despite its biological significance, menstruation continues to be surrounded by silence, stigma, and misconceptions, particularly in low-resource settings such as urban slums [1,2]. According to recent global guidelines, including the WHO/UNICEF Joint Monitoring Programme (2023) on menstrual health, equitable access to information, hygienic products, and private sanitation facilities is a fundamental human right and essential for gender equality [3]. These socio-cultural barriers, combined with poor living conditions, inadequate sanitation, and limited awareness, contribute to poor menstrual hygiene practices among adolescent girls, exposing them to a range of physical, mental, and social health risks [4,5].

Adolescence is a critical period in a girl's life, characterized by rapid physical, emotional, and social changes. The onset of menarche marks a significant milestone during this phase, and how girls manage their menstruation can have long-term implications on their confidence, school attendance, and health [6]. Inadequate menstrual hygiene management (MHM) practices-such as the use of unclean absorbents, infrequent changing, and improper disposal methods-are associated with reproductive tract infections, urinary tract infections, and, in the long term, infertility. Additionally, the lack of access to safe and private sanitation facilities often compels girls to stay at home during menstruation, negatively affecting their education [7].

Urban slums, despite being situated within cities, frequently suffer from acute deprivation of basic amenities, including water supply, toilet facilities, and healthcare services. Adolescent girls living in these areas face the dual burden of poverty and neglect, severely limiting their ability to practice safe menstrual hygiene. With low maternal literacy, limited exposure to health education, and a lack of open dialogue about menstruation, many girls depend on peers or unverified sources for information, often perpetuating myths and misconceptions [8]

In India, while the government has introduced several initiatives such as the Menstrual Hygiene Scheme (MHS) and the provision of subsidized sanitary pads under the Rashtriya Kishor Swasthya Karyakram (RKSK), the reach and effectiveness of these programs remain inconsistent, especially in urban informal settlements. Furthermore, cultural taboos surrounding menstruation discourage adolescent girls from seeking timely help and guidance [1,2,5].

Although numerous studies have assessed menstrual hygiene among school-going adolescent girls [1-5], there is limited community-based evidence specifically targeting teenage girls in urban slum populations. This knowledge gap persists despite these communities having distinct socio-economic, infrastructural, and cultural constraints that can affect MHM practices. Addressing this gap is essential to designing interventions that align with national health priorities and international menstrual health standards.

Therefore, this study aimed to assess menstrual hygiene practices and identify the socio-demographic and knowledge-related determinants of good menstrual hygiene among adolescent girls residing in urban slums of India.

## Materials and methods

Study design and setting

This community-based cross-sectional study was conducted to assess menstrual hygiene practices and identify their determinants among adolescent girls residing in urban slums of Datia, Madhya Pradesh, India. The selected study areas were officially recognized slum settlements under the local municipal authority, characterized by high population density, poor living conditions, inadequate sanitation infrastructure, and limited access to health education services.

Study duration and population

Data was collected over 12 months from April 2024 to April 2025 following administrative approvals. The study population comprised adolescent girls aged 10 to 19 years who had attained menarche and had resided in the selected slum areas for at least the previous six months. Participants were approached in their homes with the assistance of Accredited Social Health Activists (ASHAs) and trained community volunteers.

Inclusion and exclusion criteria

Girls were eligible to participate if they met the following criteria: aged between 10 and 19 years, had experienced menarche, had lived in the selected slum areas for at least six months, and provided informed assent and parental or guardian consent. Girls who had not yet attained menarche, those unwilling to participate or whose guardians did not consent, and those with chronic illnesses or cognitive impairments that could affect comprehension were excluded from the study.

Sample size and sampling technique

The sample size was calculated using the standard formula for cross-sectional studies:

\[ n = \frac{Z^2 \cdot p \cdot q}{d^2} \]

Where n = required sample size, Z = 1.96 for 95% confidence level, p = estimated prevalence of good menstrual hygiene practices [4], q = 100 - p = 50, d = absolute precision = 5%

Substituting the values; 

\[ n = \frac{(1.96)^2 \cdot 0.5 \cdot 0.5}{(0.05)^2} = \frac{3.8416 \cdot 0.25}{0.0025} = \frac{0.9604}{0.0025} = 384.16 \]

To adjust for non-response and incomplete data, a 5% buffer was added.

\[ n_{\text{final}} = 384 + (0.05 \times 384) = 384 + 19.2 = 403.2 \approx 400 \]

Hence, the final sample size was set at 400 adolescent girls.

A multistage sampling method was employed. In the first stage, five slum areas were randomly selected from the list of registered slums in Datia using the lottery method. In the second stage, households within each slum were chosen through systematic random sampling, with every nth household selected according to the sampling interval. In households with more than one eligible adolescent girl, one was randomly chosen using a chit-drawing method.

Data collection tool

A semi-structured, interviewer-administered questionnaire was used for data collection. The tool was developed after a thorough review of similar studies and was contextualized to suit the local socio-cultural environment. It was prepared in both English and the local vernacular. The questionnaire included sections on socio-demographic characteristics (age, education, family income, parental education, household sanitation), menstrual history (age at menarche, cycle duration, regularity, and associated symptoms), menstrual hygiene practices (type of absorbent used, frequency of change, washing and drying methods, disposal practices), and knowledge or awareness regarding menstruation (sources of information and understanding of safe hygiene practices).

The operational definition of good menstrual hygiene practice was defined a priori for the study as the consistent use of clean absorbents (preferably sanitary pads or clean cloth), changing absorbents at least three times per day during menstruation, washing the genital area with soap and water, proper drying of reusable cloths in sunlight, and safe disposal of menstrual waste.

The questionnaire underwent content validation by a panel of five subject experts in public health, gynecology, and adolescent health. The reliability of the tool was assessed during a pilot test on 20 teenage girls from a non-study slum area, and internal consistency was calculated using Cronbach's alpha (α=0.82), indicating good reliability. Modifications were made based on pilot feedback to improve clarity and cultural appropriateness.

Bias minimization and blinding

To reduce interviewer bias, data collectors were not informed about the specific hypotheses of the study and were instructed to maintain a neutral tone throughout the interview. Standardized scripts were used for all questions. Respondent privacy was ensured during interviews to minimize social desirability bias. Supervisors conducted random cross-checks to verify responses.

Training and quality assurance

Female data collectors-preferably those with qualifications in public health or nursing-were trained over two days on the study objectives, ethical considerations, confidentiality, and standardized methods of questionnaire administration. Regular field supervision and spot checks were conducted to ensure adherence to study protocol and data quality standards.

Data management and statistical analysis

All data were entered into Microsoft Excel (Microsoft, Redmond, Washington) and analyzed using SPSS version 26 (IBM Inc., Armonk, New York). Descriptive statistics such as frequencies, percentages, means, and standard deviations were used to summarize the variables. Bivariate associations between menstrual hygiene practices and independent variables were assessed using the Chi-squared test. Multivariate logistic regression analysis was conducted to identify significant predictors of good menstrual hygiene practices. A p-value of <0.05 was considered statistically significant.

## Results

Table 1 presents the socio-demographic characteristics of the participants. The majority were aged 14-16 years, comprising 188 (47.0%) respondents, followed by 116 (29.0%) aged 17-19 years and 96 (24.0%) aged 10-13 years. Most participants were currently attending school, accounting for 272 (68.0%), while 128 (32.0%) had either dropped out or never attended. Regarding mothers' education, 160 (40.0%) had completed primary education, 140 (35.0%) were illiterate, and 100 (25.0%) had attained secondary education or higher. A total of 240 (60.0%) participants reported a nuclear family structure, while 160 (40.0%) belonged to joint or extended families. Toilet facilities were available at home for 280 (70.0%) participants, whereas 120 (30.0%) reported having no toilet or relying on shared facilities.

**Table 1:** Socio-demographic characteristics of participants (N=400)

Variable	Category	Frequency (n)	Percentage (%)
Age group (years)	10-13	96	24.0
	14-16	188	47.0
	17-19	116	29.0
Education status	Currently attending school	272	68.0
	Dropped out/never attended	128	32.0
Mother's education	Illiterate	140	35.0
	Primary	160	40.0
	Secondary and above	100	25.0
Type of family	Nuclear	240	60.0
	Joint/extended	160	40.0
Toilet facility at home	Available	280	70.0
	Not available/shared	120	30.0

Table 2 outlines the menstrual hygiene practices of the participants. Most girls reported using sanitary pads as their primary absorbent material, with 328 (82.0%) doing so, while 72 (18.0%) used cloth or other alternatives. Regarding the frequency of absorbent change per day, 246 (61.5%) reported changing absorbents three or more times daily, whereas 154 (38.5%) changed less frequently. Only 188 (47.0%) reported washing their genital area with soap, while 212 (53.0%) used only water or did not clean the area. Safe disposal practices, such as wrapping and placing used absorbents in bins, were followed by 224 (56.0%), whereas 176 (44.0%) resorted to unsafe methods such as open disposal, burning, or flushing. A total of 150 (37.5%) girls reported school absenteeism during menstruation, while 250 (62.5%) did not miss school due to menstruation.

**Table 2:** Menstrual hygiene practices among adolescent girls

Practice	Category	Frequency (n)	Percentage (%)
Absorbent used	Sanitary pad	328	82.0
	Cloth/other	72	18.0
Change of absorbent per day	≥3 times	246	61.5
	<3 times	154	38.5
Washing genital area with soap	Yes	188	47.0
	No (only water or none)	212	53.0
Safe disposal of absorbents	Yes (wrapped and binned)	224	56.0
	No (open/burned/thrown in drain)	176	44.0
School absenteeism during menstruation	Yes	150	37.5
	No	250	62.5

Table 3 presents awareness and sources of information related to menstruation. Before experiencing menarche, 230 (57.5%) participants were aware of menstruation, while 170 (42.5%) had no prior knowledge. The primary source of information was mothers, as reported by 172 (43.0%) girls, followed by friends (84, 21.0%), teachers (56, 14.0%), and media or the internet (48, 12.0%). Notably, 40 (10.0%) participants reported having no source of information before their first menstrual experience.

**Table 3:** Knowledge and awareness related to menstruation

Knowledge Variable	Category	Frequency (n)	Percentage (%)
Aware before menarche	Yes	230	57.5
	No	170	42.5
Source of information	Mother	172	43.0
	Friends	84	21.0
	Teachers	56	14.0
	Media/Internet	48	12.0
	None	40	10.0

Table 4 shows the association between socio-demographic factors and good menstrual hygiene practices among adolescent girls (N=400). Among those whose mothers had secondary education or above, 76 (76.0%) practiced good hygiene compared to 24 (24.0%) with poor hygiene (χ²=22.18, p<0.001). Girls with access to a private toilet at home were more likely to follow good practices, with 178 (63.5%) demonstrating good hygiene versus 102 (36.5%) with poor hygiene (χ²=14.27, p<0.001). Awareness before menarche was associated with better hygiene, as 154 (66.8%) of those aware had good practices compared to 76 (33.2%) with poor practices (χ²=10.14, p=0.001). Similarly, school-going girls reported better hygiene, with 168 (61.8%) practicing good hygiene and 104 (38.2%) practicing poor hygiene (χ²=4.21, p=0.040).

**Table 4:** Association between socio-demographic factors and good menstrual hygiene practices Good menstrual hygiene practice was defined as the use of sanitary pads or clean absorbents, changing absorbents at least three times per day, cleaning the genital area with soap and water, and disposing of used absorbents safely (e.g., wrapping and placing in bins or burning hygienically). Poor menstrual hygiene practice does not meet one or more of these criteria.

Variable	Good practice (n=218)	Poor practice (n=182)	Chi-squared (χ²)	p-value
Mother's education ≥ secondary	76	24	22.18	<0.001
Private toilet at home	178	102	14.27	<0.001
Aware before menarche	154	76	10.14	0.001
School-going status	168	104	4.21	0.040

Table 5 presents the multivariate logistic regression analysis results identifying predictors of good menstrual hygiene practices. Girls whose mothers had secondary education or higher were 2.63 times more likely to practice good menstrual hygiene (adjusted odds ratio (aOR)=2.63; 95% confidence interval (CI): 1.59-4.34; p<0.001). Awareness before menarche was also a significant predictor, with those aware having nearly twice the odds of good practice (aOR=1.91; 95% CI: 1.23-2.98; p=0.004). Availability of a private toilet at home was associated with increased odds of good hygiene (aOR=2.08; 95% CI: 1.31-3.30; p=0.002). Although school-going status showed a positive association (aOR=1.48), it did not reach statistical significance (95% CI: 0.96-2.29; p=0.072).

**Table 5:** Multivariate logistic regression for determinants of good menstrual hygiene practices

Predictor variable	Adjusted odds ratio (aOR)	95% confidence interval (CI)	p-value
Mother's education ≥ secondary	2.63	1.59 - 4.34	<0.001
Awareness before menarche	1.91	1.23 - 2.98	0.004
Private toilet facility	2.08	1.31 - 3.30	0.002
School-going status	1.48	0.96 - 2.29	0.072

## Discussion

Our study demonstrates that maternal education, pre-menarcheal awareness, and in-house sanitation are the three strongest determinants of safe menstrual hygiene practices in urban slums. These findings align with recent literature, reinforcing their importance in public health planning [6,7].

Girls whose mothers had received secondary education or higher were 2.6 times more likely to practice good hygiene (adjusted odds ratio (aOR)=2.63). A similar association was observed in an urban-slum study from Jaipur, where maternal education of secondary level or above was linked with improved hygiene practices (AOR=2.19) and greater knowledge (AOR=1.46) [1]. Comparable effect sizes have also been reported internationally; a school-based survey in Ethiopia found AORs ranging from 2.7 to 3.3 for the association between maternal education and good menstrual hygiene practices [8]. These consistent findings across diverse socio-cultural settings highlight the intergenerational benefits of female literacy. Educated mothers are better positioned to challenge menstrual myths, purchase commercial absorbents, and model hygienic behaviours, thereby enabling their daughters to adopt safer practices.

Pre-menarcheal awareness also emerged as a significant determinant. In our study, nearly 58% of participants had prior knowledge about menstruation before menarche, and this awareness nearly doubled the odds of safe menstrual hygiene (aOR=1.91). Similar patterns have been reported across India. In the slums of Kolkata, 57% of girls had heard of menstruation beforehand, which was associated with increased pad usage and safer disposal habits [9]. In Odisha, a mixed-methods survey found "information before menarche" to be a significant predictor of hygienic practices (AOR=2.51) [10]. These findings support the need for early educational interventions targeting girls in upper primary school. Preparing girls for menstruation before their first period can help reduce fear, confusion, and unhygienic behaviours.

Access to private sanitation facilities was another key factor. In our study, girls with a private toilet at home were over twice as likely to practice good hygiene (aOR=2.08). Comparable findings have been reported in Odisha (AOR=2.10) and Ethiopia (AOR=2.4 for private latrine users) [10,11]. These results underscore the importance of Water, Sanitation, and Hygiene (WASH) infrastructure in supporting safe menstrual management. Even when sanitary pads are accessible, the absence of private toilets, water, or disposal mechanisms can prevent hygienic practices.

Although school-going girls had 1.48 times higher odds of practicing good hygiene, this association did not reach statistical significance in multivariable analysis (p=0.072). Studies in Jaipur and Odisha suggest that the quality of school WASH facilities-rather than enrolment alone-plays a more direct role in shaping menstrual hygiene behaviour [1,10, 12]. Schools with clean toilets, disposal bins, and regular health education sessions are associated with lower absenteeism and better practices. Our borderline result may reflect substandard sanitation in some surveyed schools, reinforcing national appeals to upgrade girl-friendly WASH facilities in urban slum schools.

Certain menstrual hygiene indicators in our study were relatively encouraging. The use of sanitary pads (82%) was higher than figures reported in Bangl (52%) and rural central India (approximately 60%) [12], possibly reflecting better market access in urban areas. However, only 61.5% of participants reported changing pads three or more times daily, far below the 80% benchmark seen in some school-based programmes. This suggests persistent barriers such as cost, limited education, or cultural taboos. Safe disposal was reported by 56% of participants, substantially higher than the 18% reported in Kolkata slums, but environmental concerns remain, as 44% still relied on burning or open dumping [9]. These contrasts reflect geographic inequalities and highlight the role of local waste management systems.

A high proportion of participants (82.0%) reported using sanitary pads as their primary absorbent material. This represents a significant improvement compared to earlier studies, such as Dasgupta and Sarkar [13], where only 11.25% reported using sanitary pads. More recent studies have found similar or slightly lower levels of pad usage, including 71.8% in Ghizer, Gilgit [14], and 74.4% in Himachal Pradesh [15]. This upward trend may reflect increased accessibility, affordability, and awareness over the past decade.

However, hygiene practices remain suboptimal in some areas. Although 61.5% of respondents reported changing their absorbents thrice daily, only 47.0% used soap to wash their genital area. Similar challenges were observed in a 2024 study from Lahore, where knowledge and practice were positively correlated (r=0.649), but gaps in practical application persisted [16]. These findings underscore the need to strengthen health education, particularly regarding cleaning practices and personal hygiene.

Of the respondents, 37.5% reported school absenteeism during menstruation, aligning with the 47.9% absenteeism rate reported in Ghizer, where girls missed an average of 2.5 days of school per month due to menstruation-related discomfort or stigma [14]. This highlights an ongoing issue that may affect educational continuity and participation in extracurricular activities during adolescence. In our study, 57.5% of participants reported prior awareness of menstruation, with mothers being the most common source of information (43.0%), followed by peers and teachers. Similar patterns have been observed in other South Asian contexts. A 2025 study in Chitral, Pakistan, noted that maternal education was a significant determinant of menstrual knowledge, particularly among minority ethnic groups [17]. Likewise, Gupta et al. reported a dominant maternal influence in rural Lucknow, India [18].

Our findings, supported by previous literature, identify three actionable drivers of safe menstrual hygiene: maternal education, early menstrual awareness, and access to private toilets. Maternal education consistently emerges as the most influential factor, with evidence from India and Ethiopia supporting its inclusion in women's literacy programmes and self-help group curricula [11]. Pre-menarcheal awareness is associated with a 1.5 to 2-fold increase in safe practices, underscoring the value of structured puberty education in grades four and five, with active involvement of mothers as peer educators. Household toilets provide a consistent two-fold advantage, reinforcing the need for targeted sanitation investments, including water and disposal provisions. While school enrolment alone may not ensure hygienic behaviour, enhancing the quality of school WASH infrastructure can reduce absenteeism and support safe menstrual management. Dedicated funding for girl-friendly toilets and regular functionality assessments, particularly in urban slum schools, is strongly recommended.

Unlike school-based surveys, our community-based study included enrolled and out-of-school adolescents - an often-overlooked population in menstrual health research. However, as with most cross-sectional designs, causal inference remains limited. The high rate of pad use observed may also be context-specific, potentially influenced by local government subsidy schemes not available in other settings like Bangladesh or Assam [12].

Limitations

This study has several limitations. As a cross-sectional design, it cannot establish causal relationships between determinants and menstrual hygiene practices. Data collection relied on self-reported information, which may be subject to recall and social desirability biases. Although the questionnaire underwent expert validation and demonstrated good internal consistency (Cronbach's alpha=0.82), responses might still be influenced by participants' willingness to disclose sensitive information. Additionally, while measures such as interviewer training, use of standardized scripts, and ensuring privacy were implemented to minimize bias, the possibility of residual measurement bias cannot be entirely ruled out. Despite these limitations, the study's strengths include its community-based approach in urban slum settings and the consideration of multiple determinants in the analysis, offering valuable insights for public health interventions.

Future longitudinal studies are needed to examine whether sustained maternal education and sanitation investments lead to long-term improvements in menstrual hygiene. Implementation research should also explore scalable interventions, such as mother-daughter workshops and community-run incinerator models, in diverse urban slum settings.

## Conclusions

This community-based cross-sectional study highlights key determinants of menstrual hygiene practices among adolescent girls in urban slums. Although most sanitary pads are used, significant gaps persisted in absorbent change frequency, genital hygiene, and safe disposal. Multivariate analysis identified maternal education, pre-menarcheal awareness, and access to private toilets as independent predictors of good practices. School enrollment showed a positive trend, but was insignificant without quality menstrual health education and supportive WASH infrastructure. Interventions should extend beyond product provision to address educational, socio-economic, and infrastructural determinants, including mother-daughter communication, school-based education, and investment in girl-friendly sanitation facilities to ensure safe, dignified menstruation and improve adolescent health and equity.

## Appendices

Questionnaire

**Table 6:** Menstrual hygiene questionnaire

Question	Response options
Section A: Socio-demographic details	
Age (in completed years)	
Class/Grade	
Religion	☐ Hindu ☐ Muslim ☐ Christian ☐ Other
Caste	☐ General ☐ OBC ☐ SC ☐ ST
Type of family	☐ Nuclear ☐ Joint ☐ Extended
Father's education	☐ Illiterate ☐ Primary ☐ Secondary ☐ Higher Secondary ☐ Graduate & above
Mother's education	☐ Illiterate ☐ Primary ☐ Secondary ☐ Higher Secondary ☐ Graduate & above
Father's occupation	
Mother's occupation	
Monthly family income	☐ < ₹5,000 ☐ ₹5,000–₹10,000 ☐ ₹10,001–₹15,000 ☐ > ₹15,000
Source of information on menstruation (tick all that apply)	☐ Mother ☐ Sister ☐ Teacher ☐ Friend ☐ TV/Media ☐ Health worker
Section B: Menstrual history	
Age at menarche	years
Duration of menstrual bleeding	days
Menstrual cycle regularity	☐ Regular ☐ Irregular
Frequency of menstrual cycle	☐ <21 days ☐ 21–35 days ☐ >35 days
Do you experience any menstrual problems?	☐ Yes ☐ No If yes, specify:
Do you attend school during menstruation?	☐ Yes ☐ No If no, reason: ☐ Pain ☐ Staining fear ☐ Lack of toilet facilities ☐ Others
Section C: Menstrual hygiene practices	
What type of absorbent do you use?	☐ Sanitary napkins ☐ Cloth (new) ☐ Cloth (old/reused) ☐ Cotton ☐ Others
How frequently do you change the absorbent in a day?	☐ Once ☐ Twice ☐ Thrice or more
Where do you dispose of used absorbents?	☐ Open space ☐ Dustbin ☐ Burn it ☐ Bury it ☐ Other
Do you wash your hands after changing the pad/cloth?	☐ Yes ☐ No If yes, with: ☐ Only water ☐ Soap and water
How do you clean your external genitalia during menstruation?	☐ Water only ☐ Soap and water ☐ Antiseptic
Frequency of washing per day	☐ Once ☐ Twice ☐ Thrice or more
Do you dry reusable cloth absorbents in sunlight?	☐ Yes ☐ No
Do you store reusable cloth in a clean place?	☐ Yes ☐ No
Section D: Menstruation-related restrictions and beliefs	
Are you restricted from any activity during menstruation?	☐ Yes ☐ No If yes, specify: ☐ Cooking ☐ Worship ☐ School ☐ Others
Do you think menstruation is a normal physiological process?	☐ Yes ☐ No
Do you feel embarrassed talking about menstruation?	☐ Yes ☐ No
Have you received health education regarding menstruation at school?	☐ Yes ☐ No
Do you feel the need for more awareness on menstrual hygiene?	☐ Yes ☐ No
